# Objective and subjective neighbourhood characteristics and suicidality: a multilevel analysis

**DOI:** 10.1017/S0033291721002579

**Published:** 2021-07-07

**Authors:** Jennifer Dykxhoorn, Joseph Hayes, Kavya Ashok, Alma Sörberg Wallin, Christina Dalman

**Affiliations:** 1 Division of Psychiatry, UCL, London, UK; 2 Department of Primary Care and Population Health, UCL, London, UK and; 3 Department of Global Public Health, Karolinska Institutet, Solna, Sweden

**Keywords:** Attempted, longitudinal studies, small-area analysis, suicidal ideation, suicide

## Abstract

**Background:**

Characteristics of the neighbourhood environment, including population density, social fragmentation, and trust, have been linked to mental health outcomes. Using a longitudinal population-based cohort, we explored the relationship between objective and subjective neighbourhood characteristics and the odds of suicidal thoughts and attempts.

**Methods:**

We conducted a longitudinal study of 20764 participants living in Stockholm County who participated in the Stockholm Public Health Survey. We used multilevel modelling to examine if suicidal thoughts and attempts were associated with neighbourhood characteristics, independent of individual associations. We included objective and subjective measures to explore if there was a different relationship between these measures of the neighbourhood environment and suicidality.

**Results:**

Associations between neighbourhood factors and suicidality were predominantly explained by individual characteristics, with the exception of neighbourhood-level deprivation and average residential trust. Each unit increase of deprivation was linked to increased odds of suicidal thoughts [Odds ratio (OR) 1.04, 95% confidence interval (CI) 1.00–1.07] and attempts (OR 1.11, 95% CI 1.06–1.17). Decreasing residential trust was associated with increased odds of suicide attempts (OR 1.09, 95% CI 1.02–1.17). There was no evidence that neighbourhood-level fragmentation or average trust in public and political institutions had an independent effect on suicidality once individual and sociodemographic factors were accounted for.

**Conclusions:**

This study showed that much of the neighbourhood-level variation in suicidal thoughts and attempts could be explained by compositional factors, including sociodemographic clustering within neighbourhoods. The independent effect of neighbourhood-level deprivation and average residential trust provide evidence that the neighbourhood context may exert an independent effect on suicidality beyond the impact of individual characteristics.

## Background

Neighbourhood-level factors, including population density, social capital, social fragmentation, and deprivation may explain some of the spatial variation in suicide risk (Zammit et al., 2014). The role of contextual factors in explaining suicidality has been of interest since Durkheim’s seminal work, where he discussed how a disconnect between the individual and societal norms may result in *anomie* and increased risk of suicide (Durkheim, 1897). Ecological associations have suggested that there may be a link between area-level factors and rates of suicidal thought, attempts, and deaths but it has not been clearly shown whether these associations can be explained by individual-level characteristics or if area-level factors exert an independent effect on suicidality (Conrad et al., 2009; Hagedoorn, Groenewegen, Roberts, & Helbich, 2020; Middleton et al., 2004; O’Farrell, Corcoran, & Perry, 2016; Rehkopf, 2006; Whitley, Gunnell, Dorling, & Smith, 1999). Multilevel studies attempt to disentangle compositional factors (e.g. individual characteristics which may be clustered within a neighbourhood) and contextual characteristics of the neighbourhood to more explore the independent effect of neighbourhood factors on suicidal thoughts and attempts (Martikainen, Mäki, & Blomgren, 2004; Qin, Agerbo, & Mortensen, 2003). One multilevel study which explored individual- and area-level deprivation in childhood and risk of later death by suicide found that most of the variance was explained by individual-level characteristics, with very little variation attributed to area-level factors (Zammit et al., 2014). O’Reilly and colleagues found similar results in Northern Ireland, where compositional factors, like gender, marital status, and socioeconomic disadvantage, accounted for most of the variance in suicide deaths (O’Reilly, Rosato, Connolly, & Cardwell, 2008). A recent study found a cross-sectional association between social cohesion and suicidal thoughts in Korean older adults (Kim & Park, 2021). Another study from Seoul found that 2.7% of the variation in rates of suicidal thoughts could be attributed to contextual factors, such as area-level organisational participation (Han & Lee, 2013). To date, few studies has used a longitudinal multilevel approach to examine if these results extend to other suicidal outcomes, including suicidal thoughts and attempts.

An additional challenge is that there are many ways to measure neighbourhood characteristics, including objective measures, such as neighbourhood deprivation, fragmentation, and physical infrastructure; and subjective factors, such as how residents feel about their neighbourhood, if they trust their neighbours, or if they feel connected to the local area. Much of the existing literature has focused on the relationship between suicidality and objectively measured neighbourhood characteristics like deprivation (Dupéré, Leventhal, & Lacourse, 2009; Rehkopf, 2006) and social fragmentation (Middleton, Gunnell, Frankel, Whitley, & Dorling, 2003). Less research has been conducted on the possible impact of subjectively reported neighbourhood characteristics on suicidal thoughts and attempts (Allen & Goldman-Mellor, 2018). Previous research on adolescents in California found that subjective perceptions of neighbourhood safety and social cohesion were associated with suicidality, but objective measures of neighbourhood quality were unrelated to adolescent suicidal outcomes (Allen & Goldman-Mellor, 2018). At the individual level, subjectively reported levels of trust in neighbours, institutions, and government have been linked to mental health outcomes (Lindstrom & Mohseni, 2009). There is some evidence that aggregated level of trust is linked to health outcomes (Engström, Mattsson, Järleborg, & Hallqvist, 2008).

To date, most studies have included a limited number of objective or subjective neighbourhood characteristics, often focusing on a single aspect of the neighbourhood environment. This has not allowed the comparison of the relative importance of subjective or objective neighbourhood characteristics.

### Aims of the study

The current study aims to further the existing literature by examining the association between multiple objective and subjective neighbourhood characteristics, and subsequent suicidal thoughts and attempts. We included objectively measured neighbourhood deprivation and fragmentation. We also explored two subjectively rated trust measures: average trust in those living in the neighbourhood and average trust in public and political institutions, as these measures may capture distinct differences; the former estimates the trust, reciprocity, and bonds between neighbours, while the later estimates the confidence in official structures and institutions.

We expected that objective and subjective factors consistent with lower neighbourhood quality and cohesion, including low levels of trust, high levels of deprivation, and high fragmentation, would be linked to increased odds of both suicidal thoughts and suicide attempts. As there was little prior evidence to indicate if objective or subjective factors were more strongly linked to suicidality, we did not develop an *a priori* hypothesis regarding the relative magnitude of effect between these ways of estimating neighbourhood characteristics.

## Methods

### Study design and population

The study population included adults (age 18–64 at baseline) or older who participated in the Stockholm Public Health Cohort (SPHC) in 2002 and who participated in at least one follow-up survey in 2006, 2010, and or 2014. We excluded individuals who did not have record of their neighbourhood at baseline, did not answer questions on trust in 2002, or did not respond to the suicide questionnaire in at least one of the follow-up waves of SPHC. This study was covered under the ethical permissions from the Regional Ethical Review Committee in Stockholm (Dnr 2010/1185-31/5).

### Outcomes

Our primary outcomes were suicidal thoughts and attempts, assessed using a self-report questionnaire administered in 2002 (baseline), 2006, 2010, and 2014. Participants were asked [translated] ‘Have you ever been in the situation that you seriously considered taking your own life, maybe even planned how you would do it?’ and ‘Have you ever made an attempt to take your life?’ Individuals who responded ‘Yes, in the last week’ or ‘Yes, in the last year’ were coded as having suicidal thoughts or suicide attempts during follow-up in order to ensure the suicidal thought and suicide attempts occurred after the baseline measure. Those who reported suicidal thoughts or attempts at baseline were excluded *(n* = 771).

### Individual-level variables

We used variables from the SPHC questionnaire and linked registers to estimate a range of individual-level exposures. To estimate an individual’s subjective view of their neighbourhood, we calculated two exposures: trust in residential area and average trust in public and political institutions. Trust in residential area was measured by the question ‘You can trust most of the people living in this neighbourhood’ with four possible response options from ‘completely agree’ to ‘completely disagree.’ Participants were also asked to rate their level of trust in public and political institutions, including the health care system, police, and politicians at local and national levels. Participants could rank their level of trust in each from (1) ‘no trust whatsoever’ to (5) ‘considerable trust.’ ‘No opinion’ was considered an equivocal response and coded as the middle response option. We summed the scores from these nine questions and rescaled them to create an ‘average trust in public and political institutions’ score between 1 and 45 (with higher value indicating higher levels of trust).

We used data from the Integrated Database for Health Insurance and Labour Market Studies (LISA) to calculate individual-level fragmentation and deprivation. Individual-level fragmentation was measured using relationship status, moving in the past year, and lone dwelling. Individual-level deprivation was measured using income quintile, education level, and employment status.

We had complete data on both individual-level objective measures (individual-level fragmentation and deprivation) for all those who had a valid address at cohort entry, however, we excluded individuals who did not have subjective measures at baseline (n = 115).

### Neighbourhood-level variables

We obtained Small Area Market Statistics (SAMS) from Statistics Sweden to estimate neighbourhood exposures. SAMS use administrative boundaries to delineate socioeconomically homogenous areas containing between 1000 and 2000 individuals (Statistika Centralbån, 2016). We merged any SAMS area with less than 50 inhabitants with the adjacent SAMS (Dykxhoorn, Lewis, Hollander, Kirkbride, & Dalman, 2020), resulting in 889 neighbourhood areas in our cohort. Those individuals did not have a valid address at baseline and thus could not be assigned a neighbourhood were excluded (*n* = 18).

For subjective neighbourhood factors, we calculated the mean scores based on individual responses from residents of each neighbourhood and calculated the average trust in the residential area and average trust in public and political institutions based on all individual responses from the neighbourhood. We then used quintiles to divide these mean scores into fifths of neighbourhood-level trust in residential area and neighbourhood-level trust in public and political institutions, from quintile 1 (very low trust) to quintile 5 (very high trust).

Objective neighbourhood characteristics were generated for each neighbourhood that contained at least one SPHC participant at baseline. These were obtained through record linkage with the LISA and the Total Population Register (RTB).

Using methods similar to previous research (Allardyce et al., 2005; Congdon, 1996) we generated a neighbourhood-level fragmentation index using three measures: neighbourhood residential mobility (proportion of people who moved in the previous year), proportion of lone dwellers, and proportion of individuals who were not married/in registered partnerships (see supplement). These indicators were z-standardised and summed to create a continuous index of fragmentation. We also used quintiles to divide neighbourhood-level fragmentation into fifths, from quintile 1 (very low fragmentation) to quintile 5 (very high fragmentation). We estimated neighbourhood-level deprivation in a similar fashion, using four measures from the LISA, including the proportion of people who were unemployed, receiving social welfare, had an income below the national median, or who had a criminal conviction. These measures were standardised and summed to create a continuous measure of neighbourhood-level deprivation. We used quintiles to divide neighbourhood-level deprivation into fifths.

### Covariates

We controlled for a range of confounders from the SPHC questionnaire including age, sex, relationship status, lone dwelling, employment, general health (GHQ), occupational classification. We included educational attainment and residential moves using information from the LISA; and region of birth, income, and population density from the RTB.

### Statistical analysis

We explored missingness within the cohort to determine if complete case analysis was appropriate. We then generated descriptive statistics of the sample. Next, using multilevel logistic regression, we investigated the association between neighbourhood characteristics at baseline and suicidality during follow-up. Individuals (level 1) were nested within neighbourhoods (level 2). We fitted a random intercept at the neighbourhood level to account for this higher-level clustering. We fitted random intercept Weibull models with normally distributed random effects to allow the baseline odds to vary across neighbourhoods. The null model, without fixed effects, was fitted to quantify the variation in the baseline odds for suicidality attributable to neighbourhood random effects. We then ran unadjusted and adjusted models and reported odds ratios (OR) with 95% confidence intervals (95% CI).

## Results

### Study sample

There were 20 764 individuals, from 889 neighbourhoods in Stockholm County (Table 1). The median number of participants in each neighbourhood was 44 (min: 1, max: 406 (IQR: 22–92). Individual levels of trust in the residential area was high, with 51.7% reporting ‘pretty much’ and 38.1% reporting ‘very much’ trust in the residential area. 14.6% of the sample had suicidal thoughts and 3.7% had attempted suicide during follow-up. Table 1 shows additional cohort characteristics.

**Fig. 1:**
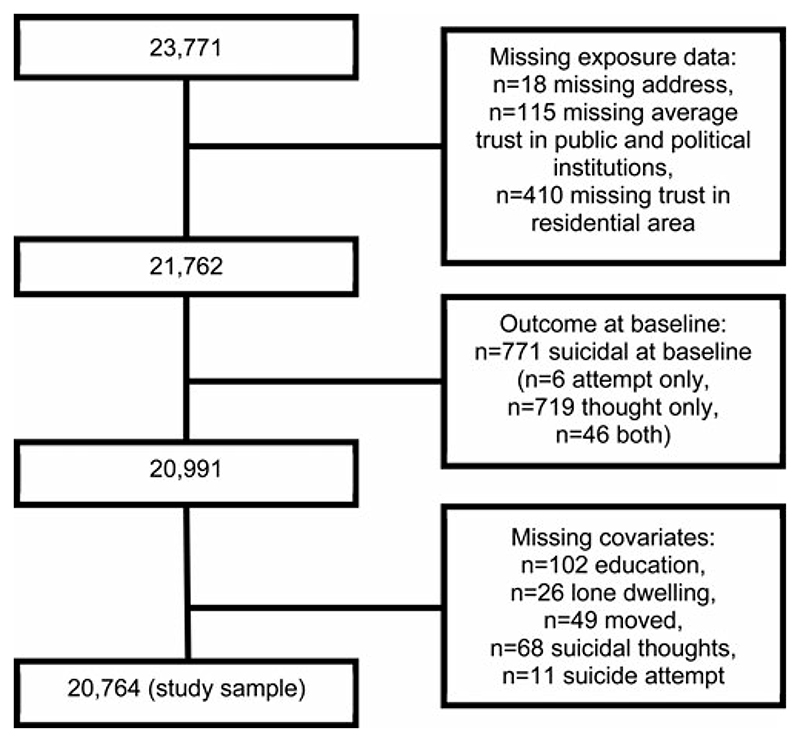
PRISMA flow chart.

We examined the full cohort and determined that the proportion of missing data was low across study variables. The highest proportion was 5.3% missing in occupational position, with all other variables having between 0.0% and 2.0% missing (Fig. 1, online Supplementary Table S1). Due to low proportions of missingness, we used complete cases analysis.

### Suicidal thoughts

#### Individual-level factors and suicidality

**Fig. 2:**
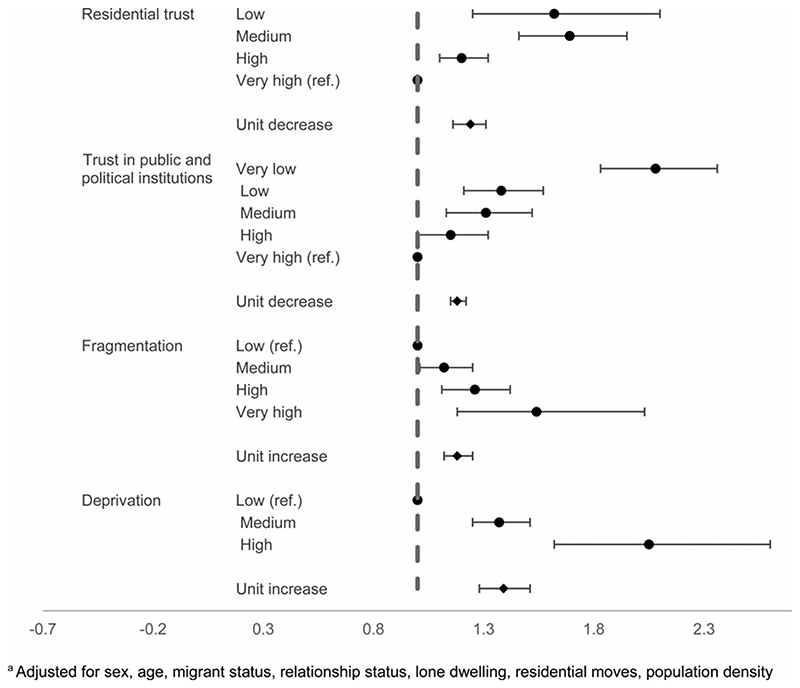
Individual-level factors and suicidal thoughts^a a^Adjusted for sex, age, migrant status, relationship status, lone dwelling, residential moves, population density

At the individual level, both objective and subjective factors were associated with increased odds of suicidal thoughts at follow-up. In the adjusted analysis, those with lower individual-level trust in the residential area had elevated odds of reporting suicidal thoughts: some trust (OR 1.20, 95% CI 1.10–1.32), low trust (OR 1.69, 95% CI 1.46–1.95), and very low trust (OR 1.62, 95% CI 1.25–2.10) compared to those with high levels of trust (Fig. 2). Each unit decrease in trust corresponded to a 24% increase in odds of reporting suicidal thoughts (95% CI 16–31). A similar pattern was found for the average trust in public and political institutions, with each unit decrease in an individual’s score on the average trust in public and political institutions corresponding to increased odds of suicidal thoughts (OR 1.18, 95% CI 1.15–1.22). Participants with higher individual-level disconnection and fragmentation from their local area (including recent residential moves, lone dwelling, and unmarried/divorced/widowed relationship status) had increased odds of suicidal thoughts. Compared to those with low fragmentation scores, individuals with higher scores had increased odds of suicidal thoughts: medium (OR 1.12, 95% CI 1.01–1.25), high (OR 1.26, 95% CI 1.11–1.42), or very high (OR 1.54, 95% CI 1.18–2.03). Individuals with high levels of deprivation, including low income, low educational attainment, and were unemployed had elevated odds of suicidal thoughts when compared to low individual-level deprivation: medium (OR 1.37, 95% CI 1.25–1.51), and high (OR 2.05, 95% CI 1.62–2.60).

#### Suicide attempts

**Fig. 3:**
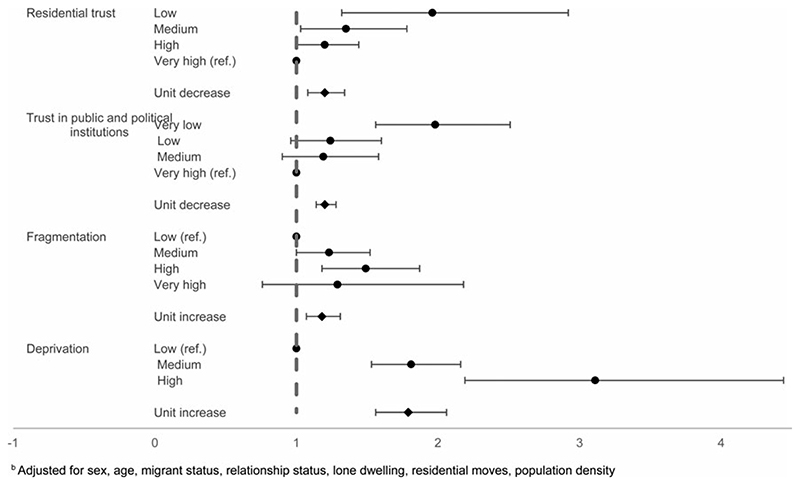
Individual-level factors and suicide attempts^b b^Adjusted for sex, age, migrant status, relationship status, lone dwelling, residential moves, population density.

There was some evidence for similar patterns of increasing odds of suicide attempts by individual-level factors. There was evidence for increased odds of suicide attempts with decreasing individual-level trust in residential area (unit decrease: OR 1.20, 95% CI 1.14–1.28) and average trust in public and political institutions (unit decrease OR 1.20, 95% CI 1.14–1.28) (online Supplementary Table S3). Individual-level fragmentation and deprivation scores were also associated with suicide attempts. For each unit increase in fragmentation, the odds of suicide attempts were 1.18 (95% CI 1.07–1.31), following adjustment for covariates. Compared to the lowest level of individual deprivation, individuals experiencing medium or high deprivation had elevated odds of suicide attempts (OR 1.81, 95% CI 1.53–2.16; OR 3.11, 95% CI 2.29–4.44, respectively) Fig. 3.

#### Neighbourhood-level factors and suicidality

**Fig. 4:**
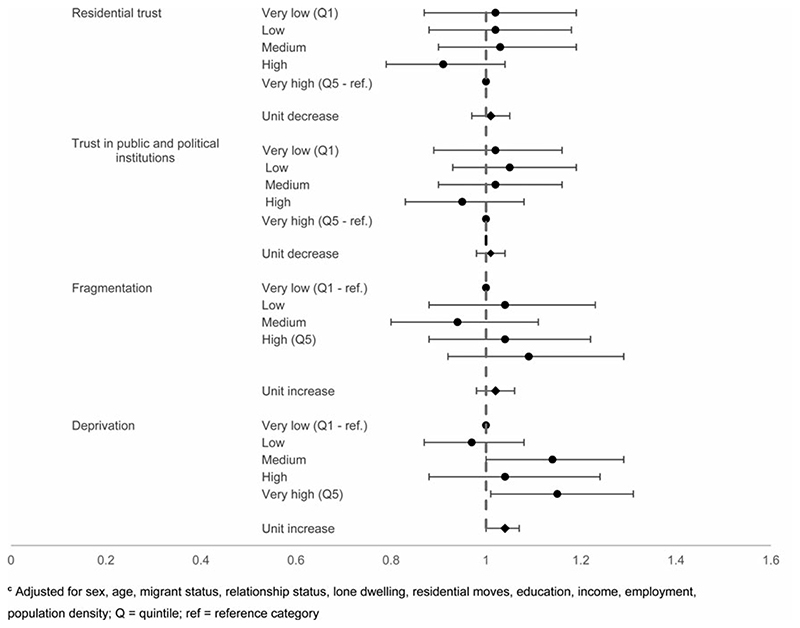
Neighbourhood-level factors and suicidal thoughts^c c^Adjusted for sex, age, migrant status, relationship status, lone dwelling, residential moves, education, income, employment, population density; Q = quintile; ref = reference category.

At the neighbourhood level, the unadjusted estimate for trust in residential area suggested a 13% increase in the odds of suicidal thoughts with each unit decrease (95% CI 10–17) (online Supplementary Table S4), but once fully adjusted for individual-level trust and sociodemographic factors, the association was null (OR 1.01, 95% CI 0.97–1.05) (Fig. 4). A weaker association was found between neighbourhood mean score on average trust in public and political institutions and suicidal thoughts in the unadjusted analysis (OR 1.03, 95% CI 1.00–1.06), which was also fully attenuated upon adjustment (OR 1.03, 95% CI 0.98–1.04).

In contrast, objectively measured neighbourhood deprivation was found to be associated with an association with suicidal thoughts following individual adjustment (unit increase: OR 1.06, 95% CI 1.03–1.09) and when fully adjusted for individual-level factors and sociodemographic factors (OR 1.04, 95% CI 1.00–1.07). Neighbourhood fragmentation suggested an association between higher levels of fragmentation and suicidal thoughts in the unadjusted estimate (OR 1.11, 95% CI 1.08–1.14), which persisted when adjusted for individual measures of fragmentation (OR 1.06, 95% CI 1.03–1.09), but attenuated once sociodemographic factors were taken into account (OR 1.02, 95% CI 0.98–1.06).

These patterns were broadly consistent with those for suicide attempts. Lower neighbourhood-level mean trust in residential areas corresponded to increased odds of suicide attempts, even following adjustment (unit decrease: OR 1.09, 95% CI 1.02–1.17), driven by high odds in the neighbourhoods with the lowest scores (very low trust: OR 1.44, 95% CI 1.05–1.95). The fully adjusted estimates did not show an association between neighbourhood-level average trust in public and political institutions score and suicide attempts, echoing what was found for suicidal thoughts.

**Fig. 5:**
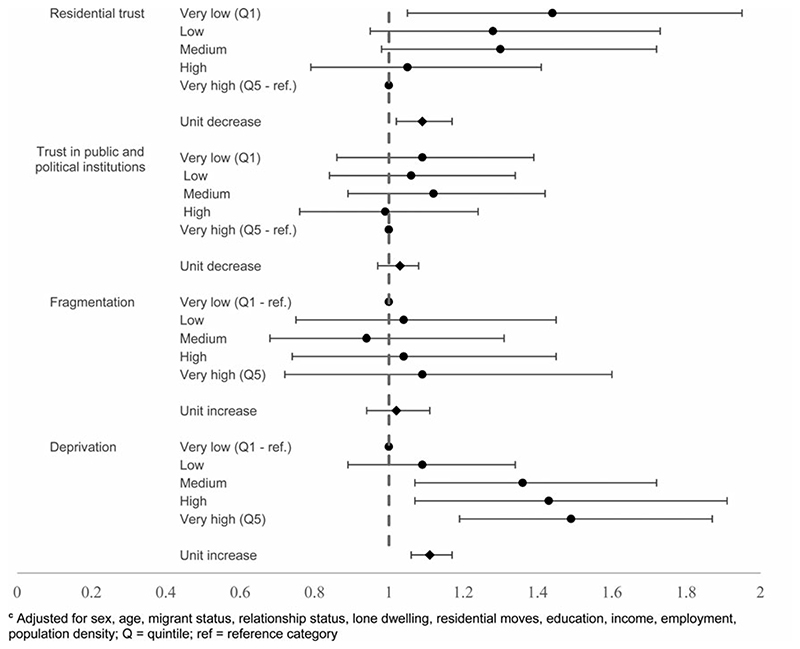
Neighbourhood-level factors and suicide attempts’^d d^Adjusted for sex, age, migrant status, relationship status, lone dwelling, residential moves, education, income, employment, population density; Q = quintile; ref = reference category.

Objectively measured neighbourhood-level deprivation was found to be related to increased suicide attempts following adjustment for individual-level deprivation and sociodemographic factors (OR 1.11, 95% CI 1.06–1.17). Neighbourhood-level fragmentation was associated with increased odds of suicide attempts in the unadjusted (OR 1.19, 95% CI 1.12–1.26) and in the individually adjusted estimate (OR 1.12, 95% CI 1.05–1.19), but these elevated odds were attenuated when fully adjusted (OR 1.07, 95% CI 0.99–1.16) (online Supplementary Table S5) Fig. 5.

## Discussion

### Principal findings

At the individual level, both objective (high deprivation and fragmentation) and subjective (low trust in residential area and trust in public and political institutions) were associated with increased odds of suicidal thoughts and attempts.

At the neighbourhood level, higher levels of deprivation were associated with increased odds of suicidal thoughts and attempts. Decreasing average trust in residential area was associated with increased odds of suicide attempts, although this was not found for suicidal thoughts. Neighbourhood-level fragmentation and average trust in public and political institutions were not associated with increased odds of suicidality once individual characteristics were considered.

### Limitations and strengths

There are several limitations that must be noted, including cohort response rates and limited information for calculating social fragmentation. Among those selected for the survey, the response rates in 2002, 2006, and 2010 were 62, 61, and 56%, respectively (Svensson et al., 2013). This study may have been impacted by selection bias, as the response rate was lower among men, migrant populations, and those with lower levels of income and education (Svensson et al., 2013). It is plausible that the response rate may have been lower for those experiencing suicidal thoughts and attempts as well as those living in more deprived areas. There may also be differential losses to follow-up over the cohort period, as those experiencing mental health problems or socioeconomic difficulties may be more likely to drop out from subsequent waves of the survey.

Although we had access to detailed information on individuals and neighbourhoods in the population registers and cohort data, we were limited to what was available in these sources, which restricted the construction of our social fragmentation indicator. In previous literature, the Congdon anomie score has been frequently used to estimate social fragmentation. This score includes four aspects: (1) % of persons who have moved in the last year, (2) % unmarried persons, (3) % single person households, and (4) % of persons in private rented accommodations (Congdon, 1996). We did not have access to information about housing tenure, so we only included three measures in our social fragmentation score. This deviation from the Congdon score may mean that these results are not directly comparable to other studies of social fragmentation.

A key strength of this analysis was the rich data available in the Stockholm Public Health Cohort and linked Swedish registers. We were able to combine self-report responses from the cohort study with register information to generate detailed estimates of exposures, outcomes, and confounders. Further, we were able to calculate area-level exposures on small, demographically homogenous areas (SAMS), which reduced the risk of exposure misclassification. Previous research on the appropriate scale for area-based analysis must balance size and power, as smaller areas tend to be more homogeneous but often lack the power to detect an effect. This has been demonstrated in a previous study, which found that the association between suicide rates and area deprivation disappeared when moving from smaller areas (wards) to larger areas (local authorities) (Rezaeian, Dunn, St. Leger, & Appleby, 2006). We measured exposures at both the individual and neighbourhood levels and used a multilevel design to clarify the relative importance of the factors at each level. This allowed us to isolate the added effect of neighbourhood characteristics, above the individual factors. We used a longitudinal design to reduce the risk of differential recall, as those experiencing suicidal thoughts or attempts may have a tendency to report lower levels of neighbourhood trust (Engström et al., 2008).

### Comparisons with previous literature

The findings at the individual level were consistent with previous research which has shown an association between suicidal thoughts or attempts and individual sociodemographic characteristics, including income (Wetherall, Daly, Robb, Wood, & O’Connor, 2015), employment (Gunnell, Harbord, Singleton, Jenkins, & Lewis, 2004), education, relationship status (Gunnell et al., 2004), lone dwelling (Gisle & Van Oyen, 2013), and residential moves (Forman-Hoffman, Glasheen, & Ridenour, 2018). There has been less research on the association of neighbourhood-level factors on suicidality. Like our study, previous research has shown different associations between deprivation and fragmentation on suicidality. Importantly, much of the existing literature uses ecological designs to explore the relationship between suicide mortality and neighbourhood characteristics, which may exhibit different patterns of association to suicidal thoughts and attempts. Previous studies have showed a relationship between neighbourhood deprivation and suicidal thoughts and attempts (Cairns, Graham, & Bambra, 2017; O’Farrell et al., 2016). One study using national mortality statistics found that suicide deaths were predicted by neighbourhood deprivation, but that fragmentation was only associated with suicide risk in specified population sub-groups (O’Farrell et al., 2016). This contrasts with earlier research which found at an ecological level that suicide was more strongly associated with fragmentation than deprivation (Whitley et al., 1999). To our knowledge, association between neighbourhood-level trust and suicidal outcomes have not been explored in previous research.

When comparing objective (deprivation and fragmentation) with subjective (residential trust and trust in public and political institutions) at the neighbourhood-level, we found differing patterns of association with suicidal thoughts and attempts. One objective measure, neighbourhood-level deprivation, was linked to both suicidal thoughts and attempts, but the other objective measure, neighbourhood-level fragmentation, was not associated with suicidality. Similarly, one subjective measure, neighbourhood-level residential trust, was found to be associated with suicide attempts, but the other subjective measure (neighbourhood level trust in public and political institutions) was not associated with suicidal thoughts or attempts. This varies from a previous cross-sectional study of adolescents, which found an association between perceived neighbourhood characteristics and suicidal thoughts and behaviours, but no evidence for a link between objective measures and suicidality (Allen & Goldman-Mellor, 2018). However, the relative import of objective and subjective factors remains equivocal and requires further exploration in longitudinal population cohorts.

### Meaning of findings

The unadjusted models suggested that some of the variance in odds of suicidal thoughts and attempts may be explained by neighbourhood factors, including neighbourhood-level residential trust, trust in public and political institutions, fragmentation, and deprivation. However, once individual scores and sociodemographic characteristics were considered only neighbourhood-level deprivation and residential trust remained associated with suicidality. Specifically, increasing neighbourhood-level deprivation was associated with increased odds of suicidal thoughts and attempts, while decreasing residential trust was associated with increased odds of suicide attempts.

The complete attenuation of the association with other neighbourhood characteristics, including neighbourhood-level fragmentation and trust in public and political institutions supports a composition rather than contextual explanation for the results. The multilevel design of this study has enabled us to begin to disentangle the contextual and compositional effects, and these findings suggest that observed neighbourhood variation in suicidality may be largely attributed to the compositional characteristics of the people living within the neighbourhood. Residential segregation by socioeconomic status is persistent in many cities, leading to clustering of individuals with similar socioeconomic status in neighbourhood areas (Pickett & Wilkinson, 2008). Thus, once the individual characteristics are taken into account, the additional effect of neighbourhood factors was marginal, suggesting that observed associations between most area-level factors and rates of suicidal thoughts and attempts can be attributed to the characteristics of the residents. However, it is possible that other neighbourhood characteristics, like availability of green or blue spaces, transit connectivity, or the density of alcohol outlets might have a larger contribution to rates of suicidality, which could be explored in future studies.

In contrast, the associations between neighbourhood-level deprivation and average residential trust following adjustment for individual and demographic factors, provide evidence for contextual effects, where area-level factors conferred additional risk beyond individual and compositional explanations. Neighbourhood-level deprivation was linked to both increased suicidal thoughts and attempts, while residential trust remained associated with elevated odds of suicide attempts, following adjustment for individual and demographic characteristics.

Living in neighbourhoods characterised by high levels of deprivation and distrust of neighbours may be linked to suicidal thoughts and attempts through entrapment or defeat, consistent with the arrested flight (Gilbert & Allan, 1998), cry of pain (Johnson, Gooding, & Tarrier, 2008), Schematic Appraisal Model of Suicide (Hagedoorn et al., 2020), and the Integrated Motivational-Volitional Model of suicidal behaviour (O’Connor & Kirtley, 2018; O’Connor & Nock, 2014). These models suggest that individuals who feel trapped by external circumstances may exhibit suicidal thoughts as they fail to find a way to escape their problems. Individuals living in deprived neighbourhoods may be more likely to feel trapped and without options for escape (Turney, Kissane, & Edin, 2013).

These results suggest that multifaceted suicide prevention activities which target high-risk individuals clustering within neighbourhoods as well as high-risk neighbourhoods, characterised by high deprivation and distrust could be useful in reducing suicidal thoughts and attempts. Before such public mental health interventions could be implemented, further understanding of the underlying mechanisms which drive the contextual effects are warranted, as is further evidence for effective areabased suicide prevention interventions.

## Conclusions

Our findings show that while much of the neighbourhood variation in suicidal thoughts and attempts can be attributed to compositional characteristics of the area, neighbourhood-level deprivation and residential trust may confer additional risk. These findings support both compositional and contextual explanations for area-level variation in suicidality. These results suggest that multifaceted suicide prevention programmes which target both high-risk individuals and high-risk neighbourhoods could be beneficial in reducing rates of suicidal thoughts and attempts.

## Supplementary Material

The supplementary material for this article can be found at https://doi.org/10.1017/S0033291721002579.

Supporting Information

## Figures and Tables

**Table 1:** Baseline characteristics from the Stockholm public health cohort

Characteristics	*n* (20 764)	%
Suicidal thoughts		
Yes	3038	14.6
No	17 726	85.4
Suicide attempt		
Yes	758	3.7
No	20 006	96.4
Sex		
Female	11 540	55.6
Male	9224	44.4
Age at baseline		
18–24	1336	6.4
25–44	7757	37.4
45–64	8348	40.2
65–84	3323	16.0
Migrant status		
Migrant	2927	14.1
Child of migrants	1987	9.6
Swedish-born to Swedish parents	13 905	67.0
Swedish-born, no registered parents	1945	9.4
Relationship status		
Married/registered partnership	10 438	50.3
Unmarried/divorced/widowed	10 326	49.7
Lone dwelling		
Lives with others	16 842	81.1
Lives alone	3922	18.9
Moved in last year		
Yes	1765	8.5
No	18 999	91.5
Educational attainment		
Compulsory education or less	36	0.2
Secondary education	11 934	57.5
Post-secondary education	8794	42.4
Family income		
Quintile 1 (lowest)	1985	9.6
Quintile 2	3255	15.7
Quintile 3	4265	20.5
Quintile 4	4058	19.5
Quintile 5 (highest)	7201	34.7
Employment status		
Unemployed	863	4.2
Employed	19 901	95.8
Average trust in public and political institutions		
Quintile 1 (lowest)	4634	22.3
Quintile 2	4985	24.0
Quintile 3	2958	14.2
Quintile 4	4538	21.9
Quintile 5 (highest)	3649	17.6
Trust in residential area		
Little	385	1.8
Not very much	1726	8.3
Pretty much	10 735	51.7
Very much	7918	38.1
Neighbourhood mean average trust in public and political institutions		
Quintile 1 (lowest)	4158	20.0
Quintile 2	4156	20.0
Quintile 3	4155	20.0
Quintile 4	4220	20.3
Quintile 5 (highest)	4075	19.6
Neighbourhood mean trust in residential area		
Quintile 1 (lowest)	4181	20.1
Quintile 2	4212	20.3
Quintile 3	4048	19.5
Quintile 4	4167	20.1
Quintile 5 (highest)	4156	20.0
Neighbourhood deprivation		
Quintile 1 (lowest)	10 022	48.3
Quintile 2	4715	22.7
Quintile 3	2405	11.6
Quintile 4	1253	6.0
Quintile 5 (highest)	2369	11.4
Neighbourhood social fragmentation		
Quintile 1 (lowest)	2256	10.9
Quintile 2	3126	15.1
Quintile 3	3350	16.1
Quintile 4	3970	19.1
Quintile 5 (highest)	8062	38.8
