# Supplementary material for: Objective and subjective neighbourhood characteristics and suicidality: a multilevel analysis
Source: Psychol Med. 2021 Jul 7;53(4):1166–75. doi: 10.1017/S0033291721002579 (PMC7614302; doi:10.1017/S0033291721002579)
Supplement: Supplementary file 1 [file S0033291721002579sup001.docx]

**SUPPLEMENTAL INFORMATION**

**Variables**

At the individual level, fragmentation was measured using relationship status, lone dwelling, and moving in the past year. Level of individual deprivation was measured using income quintile, education level, and employment status.

*Individual-level deprivation*

We created dichotomous variables of income (low income vs. not low income), employment (unemployed vs. employed), and education (primary education vs. secondary education or higher). These were summed to create an individual level of deprivation.

*Individual-level fragmentation*

We used summed individual responses to the three measures of fragmentation: relationship status, lone dwelling, and residential moves. We used relationship status as recorded in the LISA in order to be consistent with those used to calculate the neighbourhood fragmentation score. When compared to the self-reported relationship status contained in SPHC, there was high agreement with only 40 (0.4%) recorded as married in the LISA indicating they were unmarried in the self-report SPHC, and 46 (0.4%) who were listed as unmarried in the LISA reported being married in SPHC. We dicotomised this to be married/registered partnership vs. unmarried, divorced, and widowed. Similarly, a dichotomous lone dwelling variable was calculated (living with others vs. lone dwelling). Residential moves were estimated by exploring if the individual was registered to the same neighbourhood as they were in the previous year. This variable was dicotomised (did not move in past year vs. moved in past year). This measure does not capture residential moves within the SAMS but would capture any moves beyond the neighbourhood boundaries. These three variables were combined to create an individual fragmentation score which was used to estimate the association between individual-level fragmentation and suicidal outcomes.

**Supplemental table 1 Proportion of missingness by study variable**

|  | **n** | **%** |
| --- | --- | --- |
| **Baseline (2002)** |  |  |
| Age | 0 | 0.0 |
| Sex | 0 | 0.0 |
| Region | 0 | 0.0 |
| Relationship status | 0 | 0.0 |
| Income | 0 | 0.0 |
| Lone dwelling | 101 | 0.4 |
| Education | 129 | 0.6 |
| Occupational position | 1,206 | 5.3 |
| GHQ score | 464 | 2.0 |
| Swedish/foreign born | 0 | 0.0 |
| **Follow-up (missing at all time points)** |  |  |
|  | 75 | 0.3 |
|  | 72 | 0.3 |
|  |  |  |

|  | **n** | **%** |
| --- | --- | --- |
| **Baseline (2002)** |  |  |
| Age | 0 | 0.0 |
| Sex | 0 | 0.0 |
| Region | 0 | 0.0 |
| Relationship status | 0 | 0.0 |
| Income | 0 | 0.0 |
| Lone dwelling | 101 | 0.4 |
| Education | 129 | 0.6 |
| Occupational position | 1,206 | 5.3 |
| GHQ score | 464 | 2.0 |
| Swedish/foreign born | 0 | 0.0 |
| **Follow-up (missing at all time points)** |  |  |
|  | 75 | 0.3 |
|  | 72 | 0.3 |
| **Follow-up (2007)** |  |  |
| Suicidal thoughts | 250 | 1.1 |
| Suicide attempts | 244 | 1.1 |
| **Follow-up (2010)** |  |  |
| Suicidal thoughts | 4,456 | 19.5 |
| Suicide attempts | 4,412 | 19.3 |
| **Follow-up (2014)** |  |  |
| Suicidal thoughts | 9,017 | 39.4 |
| Suicide attempts | 8,984 | 39.3 |

**Supplemental table 2 Individual-level factors and suicidal thoughts**

|  | | n | | % | | Unadjusted | | | | | | | Adjusted | | | | | |  |
| --- | --- | --- | --- | --- | --- | --- | --- | --- | --- | --- | --- | --- | --- | --- | --- | --- | --- | --- | --- |
|  | | (cases) | |  | | OR | | 95% | | CI | | OR | | | 95% | | CI |  |  |
| **SUBJECTIVE** | |  | |  | |  | |  | |  | |  | | |  | |  |  |  |
| **Trust in residential area ^a^** | |  | |  | |  | |  | |  | |  | | |  | |  |  |  |
| Disagree completely (low trust) | | 86 | | 22.3 | | **2.15** | | **1.68** | | **2.77** | | **1.62** | | | **1.25** | | **2.10** |  |  |
| Disagree to some extent | | 389 | | 12.8 | | **2.18** | | **1.90** | | **2.49** | | **1.69** | | | **1.46** | | **1.95** |  |  |
| Agree to some extent | | 1,638 | | 15.3 | | **1.35** | | **1.24** | | **1.48** | | **1.20** | | | **1.10** | | **1.32** |  |  |
| Completely agree (high trust) | | 925 | | 11.7 | | 1 | |  | |  | | 1 | | |  | |  |  |  |
|  | |  | |  | |  | |  | |  | |  | | |  | |  |  |  |
| Unit decrease | | 3,038 | | 14.6 | | **1.39** | | **1.32** | | **1.47** | | **1.24** | | | **1.16** | | **1.31** |  |  |
|  |  | |  | |  | |  | |  | |  | | |  | |  | | |  |
| **Average trust in public and political institutions ^a^** | | | | | | | | | |  | |  | | |  | |  |  |  |
| Very low | | 935 | | 20.2 | | **2.02** | | **1.78** | | **2.30** | | **2.08** | | | **1.83** | | **2.36** |  |  |
| Low | | 716 | | 14.4 | | **1.34** | | **1.18** | | **1.53** | | **1.38** | | | **1.21** | | **1.57** |  |  |
| Medium | | 415 | | 14.0 | | **1.31** | | **1.13** | | **1.52** | | **1.31** | | | **1.13** | | **1.52** |  |  |
| High | | 566 | | 12.5 | | 1.14 | | 0.99 | | 1.30 | | **1.15** | | | **1.00** | | **1.32** |  |  |
| Very high | | 406 | | 11.1 | | 1 | |  | |  | | 1 | | |  | |  |  |  |
|  | |  | |  | |  | |  | |  | |  | | |  | |  |  |  |
| Unit decrease | | 3,038 | | 14.6 | | **1.18** | | **1.15** | | **1.21** | | **1.18** | | | **1.15** | | **1.22** |  |  |
| **OBJECTIVE** | |  | |  | |  | |  | |  | |  | | |  | |  |  |  |
| **Fragmentation ^b^** | |  | |  | |  | |  | |  | |  | | |  | |  |  |  |
| Low | | 1,111 | | 11.3 | | 1 | |  | |  | | 1 | | |  | |  |  |  |
| Medium | | 1,042 | | 16.7 | | **1.56** | | **1.42** | | **1.71** | | **1.12** | | | **1.01** | | **1.25** |  |  |
| High | | 800 | | 18.3 | | **1.74** | | **1.58** | | **1.93** | | **1.26** | | | **1.11** | | **1.42** |  |  |
| Very high | | 85 | | 24.6 | | **2.53** | | **1.96** | | **3.26** | | **1.54** | | | **1.18** | | **2.03** |  |  |
|  | |  | |  | |  | |  | |  | |  | | |  | |  |  |  |
| Unit decrease | | 3,038 | | 14.6 | | **1.34** | | **1.28** | | **1.40** | | **1.13** | | | **1.07** | | **1.20** |  |  |
|  |  | |  | |  | |  | |  | |  | | |  | |  | | |  |
| **Deprivation ^c^** | |  | |  | |  | |  | |  | |  | | |  | |  |  |  |
| Low | | 1,903 | | 12.7 | | **1** | |  | |  | | **1** | | |  | |  |  |  |
| Medium | | 1,029 | | 19.0 | | **1.60** | | **1.48** | | **1.74** | | **1.37** | | | **1.25** | | **1.51** |  |  |
| High | | 106 | | 29.0 | | **2.77** | | **2.19** | | **3.49** | | **2.05** | | | **1.62** | | **2.60** |  |  |
|  | |  | |  | |  | |  | |  | |  | | |  | |  |  |  |
| Unit decrease | | 3,038 | | 14.6 | | **1.62** | | **1.51** | | **1.74** | | **1.39** | | | **1.28** | | **1.51** |  | |
|  | |  | |  | |  | |  | |  | |  | | |  | |  |  |  |

^a^ Adjusted for sex, age, migrant status, relationship status, lone dwelling, residential moves, education, income, employment status, population density

^b^ Adjusted for sex, age, migrant status, education, income, employment status, population density

^c^ Adjusted for sex, age, migrant status, relationship status, lone dwelling, residential moves, population density

**Supplemental table 3 Individual-level factors and suicide attempts**

|  | n | | % | | Unadjusted | | | | Adjusted | | | | | | |
| --- | --- | --- | --- | --- | --- | --- | --- | --- | --- | --- | --- | --- | --- | --- | --- |
|  | (cases) | |  | | OR | 95% | CI | | OR | | 95% | | CI | | |
| **SUBJECTIVE** |  | |  | |  |  |  | |  | |  | |  | | |
| **Trust in residential area ^a^** | |  | |  |  |  | |  | |  | |  | |  |  |
| Disagree completely (low trust) | | 36 | | 9.4 | **3.98** | **2.73** | **5.78** | | | **1.96** | | **1.32** | | **2.92** |  |
| Disagree to some extent | | 102 | | 5.9 | **2.42** | **1.89** | **3.11** | | | **1.35** | | **1.03** | | **1.78** |  |
| Agree to some extent | | 422 | | 3.9 | **1.59** | **1.34** | **1.89** | | | **1.20** | | **1.00** | | **1.44** |  |
| Completely agree (high trust) | | 198 | | 2.5 | 1 |  |  | | | 1 | |  | |  |  |
|  | |  | |  |  |  |  | | |  | |  | |  |  |
| Unit decrease | | 758 | | 3.6 | **1.57** | **1.42** | **1.73** | | | **1.20** | | **1.08** | | **1.34** |  |
|  | |  | |  |  |  |  | | |  | |  | |  |  |
| **Average trust in public and political institutions ^a^** | | | | |  |  |  | | |  | |  | |  |  |
| Very low | | 265 | | 5.7 | **2.12** | **1.68** | **2.67** | | | **1.98** | | **1.56** | | **2.51** |  |
| Low | | 175 | | 3.5 | 1.27 | 0.99 | 1.63 | | | 1.24 | | 0.96 | | 1.60 |  |
| Medium | | 102 | | 3.4 | 1.25 | 0.94 | 1.65 | | | 1.19 | | 0.90 | | 1.58 |  |
| High | | 114 | | 2.5 | 0.90 | 0.69 | 1.18 | | | 0.90 | | 0.68 | | 1.18 |  |
| Very high | | 102 | | 2.8 | 1 |  |  | | | 1 | |  | |  |  |
|  | |  | |  |  |  |  | | |  | |  | |  |  |
| Unit decrease | | 758 | | 3.6 | **1.23** | **1.17** | **1.30** | | | **1.20** | | **1.14** | | **1.28** |  |
| **OBJECTIVE** | |  | |  |  |  |  | | |  | |  | |  |  |
| **Fragmentation** ^b^ |  | |  | |  |  |  | |  | |  | |  | | |
| Low | 229 | | 2.3 | | 1 |  |  | | 1 | |  | |  | | |
| Medium | 270 | | 4.3 | | **1.88** | **1.57** | **2.25** | | **1.23** | | **1.00** | | **1.52** | | |
| High | 241 | | 5.5 | | **2.42** | **2.01** | **2.92** | | **1.49** | | **1.18** | | **1.87** | | |
| Very high | 18 | | 5.2 | | **2.28** | **1.39** | **3.74** | | 1.29 | | 0.76 | | 2.18 | | |
|  |  | |  | |  |  |  | |  | |  | |  | | |
| Unit decrease | 758 | | 3.6 | | **1.49** | **1.37** | **1.62** | | **1.18** | | **1.07** | | **1.31** | | |
|  |  | |  | |  |  |  | |  | |  | |  | | |
| **Deprivation ^c^** |  | |  | |  |  |  | |  | |  | |  | | |
| Low | 401 | | 2.7 | | 1 |  |  | | 1 | |  | |  | | |
| Medium | 316 | | 5.8 | | **2.25** | **1.93** | **2.61** | | **1.81** | | **1.53** | | **2.16** | | |
| High | 41 | | 11.2 | | **4.55** | **3.23** | **6.40** | | **3.11** | | **2.19** | | **4.44** | | |
|  |  | |  | |  |  |  | |  | |  | |  | | |
| Unit decrease | 758 | | 3.6 | | **2.20** | **1.94** | **2.49** | | **1.79** | | **1.56** | | **2.06** | | |
|  |  | |  | |  |  |  | |  | |  | |  | | |

^a^ Adjusted for sex, age, migrant status, relationship status, lone dwelling, residential moves, education, income, employment status, population density

^b^ Adjusted for sex, age, migrant status, education, income, employment status, population density

^c^ Adjusted for sex, age, migrant status, relationship status, lone dwelling, residential moves, population density

**Supplemental table 4 Neighbourhood-level factors and suicidal thoughts**

|  | n (cases) | % | Unadjusted | | | Individually-adjusted | | | Fully adjusted | | |
| --- | --- | --- | --- | --- | --- | --- | --- | --- | --- | --- | --- |
|  |  |  | OR | 95% | CI | OR | 95% | CI | OR | 95% | CI |
| **SUBJECTIVE** |  |  |  |  |  |  |  |  |  |  |  |
| **Trust in residential area** ^a^ | | |  |  |  |  |  |  |  |  |  |
| Very low (Q1) | 744 | 17.8 | **1.57** | **1.38** | **1.78** | **1.23** | **1.07** | **1.41** | 1.02 | 0.87 | 1.19 |
| Low | 682 | 16.2 | **1.40** | **1.23** | **1.59** | **1.20** | **1.05** | **1.38** | 1.02 | 0.88 | 1.18 |
| Medium | 636 | 15.0 | **1.28** | **1.12** | **1.46** | **1.16** | **1.02** | **1.33** | 1.03 | 0.90 | 1.19 |
| High | 488 | 11.9 | 0.98 | 0.85 | 1.12 | 0.93 | 0.81 | 1.07 | 0.91 | 0.79 | 1.04 |
| Very high (Q5) | 488 | 12.1 | 1 |  |  | 1 |  |  | 1 |  |  |
|  |  |  |  |  |  |  |  |  |  |  |  |
| Unit decrease | 3,038 | 14.6 | **1.13** | **1.10** | **1.17** | **1.07** | **1.04** | **1.11** | 1.01 | 0.97 | 1.05 |
|  |  |  |  |  |  |  |  |  |  |  |  |
| **Average trust in public and political institutions** ^b^ | | | | |  |  |  |  |  |  |  |
| Very low (Q1) | 624 | 15.0 | 1.09 | 0.96 | 1.25 | 0.96 | 0.84 | 1.10 | 1.02 | 0.89 | 1.16 |
| Low | 654 | 15.7 | **1.16** | **1.01** | **1.33** | 1.07 | 0.93 | 1.23 | 1.05 | 0.93 | 1.19 |
| Medium | 612 | 14.7 | 1.08 | 0.94 | 1.24 | 1.02 | 0.88 | 1.17 | 1.02 | 0.90 | 1.16 |
| High | 570 | 13.5 | 0.98 | 0.85 | 1.13 | 0.94 | 0.81 | 1.09 | 0.95 | 0.83 | 1.08 |
| Very high (Q5) | 578 | 14.2 | 1 |  |  | 1 |  |  | 1 |  |  |
|  |  |  |  |  |  |  |  |  |  |  |  |
| Unit decrease | 3,038 | 14.6 | **1.03** | **1.00** | **1.06** | 1.00 | 0.97 | 1.03 | 1.01 | 0.98 | 1.04 |
|  |  |  |  |  |  |  |  |  |  |  |  |
| **OBJECTIVE** |  |  |  |  |  |  |  |  |  |  |  |
| **Fragmentation ^c^ index** ^c^ |  |  |  |  |  |  |  |  |  |  |  |
| Very low (Q1) | 274 | 12.2 | 1 |  |  | 1 |  |  | 1 |  |  |
| Low | 409 | 13.1 | 1.08 | 0.91 | 1.28 | 1.06 | 0.90 | 1.26 | 1.04 | 0.88 | 1.23 |
| Medium | 419 | 12.5 | 1.03 | 0.87 | 1.22 | 0.98 | 0.83 | 1.15 | 0.94 | 0.80 | 1.11 |
| High | 581 | 14.6 | **1.26** | **1.08** | **1.49** | 1.13 | 0.96 | 1.32 | 1.04 | 0.88 | 1.22 |
| Very high (Q5) | 1,355 | 16.8 | **1.47** | **1.27** | **1.70** | **1.24** | **1.07** | **1.44** | 1.09 | 0.92 | 1.29 |
|  |  |  |  |  |  |  |  |  |  |  |  |
| Unit increase | 3,038 | 14.6 | **1.11** | **1.08** | **1.14** | **1.06** | **1.03** | **1.09** | 1.02 | 0.98 | 1.06 |
|  |  |  |  |  |  |  |  |  |  |  |  |
| **Deprivation ^d^ index** ^d^ |  |  |  |  |  |  |  |  |  |  |  |
| Very low (Q1) | 1,326 | 13.2 | 1 |  |  | 1 |  |  | 1 |  |  |
| Low | 670 | 14.2 | 1.08 | 0.97 | 1.21 | 1.02 | 0.92 | 1.13 | 0.97 | 0.87 | 1.08 |
| Medium | 412 | 17.1 | **1.34** | **1.18** | **1.53** | **1.20** | **1.06** | **1.36** | **1.14** | **1.00** | **1.29** |
| High | 197 | 15.7 | **1.24** | **1.04** | **1.48** | 1.11 | 0.94 | 1.31 | 1.04 | 0.88 | 1.24 |
| Very high (Q5) | 433 | 18.3 | **1.47** | **1.29** | **1.67** | **1.26** | **1.11** | **1.43** | **1.15** | **1.01** | **1.31** |
|  |  |  |  |  |  |  |  |  |  |  |  |
| Unit increase | 3,038 | 14.6 | **1.10** | **1.07** | **1.14** | **1.06** | **1.03** | **1.09** | **1.04** | **1.00** | **1.07** |
|  |  |  |  |  |  |  |  |  |  |  |  |

^a^ Individually-adjusted: trust in residential area; Fully-adjusted: trust in residential area, sex, age, migrant status, relationship status, lone dwelling, residential moves, education, income, employment, population density

^b^ Individually-adjusted: average trust in public and political institutions; Fully-adjusted: average trust in public and political institutions, sex, age, migrant status, relationship status, lone dwelling, residential moves, education, income, employment, population density

^c^ Individually-adjusted: relationship status, lone dwelling, residential moves; Fully-adjusted: sex, age, migrant status, relationship status, lone dwelling, residential moves, education, income, employment, population density

^d^ Individually-adjusted: income, education; Fully-adjusted: sex, age, migrant status, relationship status, lone dwelling, residential moves, education, income, employment, population density

Q = quintile

**Supplemental table 5 Neighbourhood-level factors and suicide attempts**

|  | n | % | Unadjusted | | | Individually-adjusted | | | Fully adjusted | | |
| --- | --- | --- | --- | --- | --- | --- | --- | --- | --- | --- | --- |
|  | (cases) |  | OR | 95% | CI | OR | 95% | CI | OR | 95% | CI |
| **SUBJECTIVE** |  |  |  |  |  |  |  |  |  |  |  |
| **Trust in residential area** ^a^ | | |  |  |  |  |  |  |  |  |  |
| Very low (Q1) | 226 | 5.4 | **2.52** | **1.97** | **3.24** | **1.91** | **1.46** | **2.50** | **1.44** | **1.05** | **1.95** |
| Low | 176 | 4.2 | **1.93** | **1.49** | **2.49** | **1.62** | **1.24** | **2.11** | 1.28 | 0.95 | 1.73 |
| Medium | 159 | 3.7 | **1.72** | **1.32** | **2.23** | **1.53** | **1.17** | **2.00** | 1.30 | 0.98 | 1.72 |
| High | 108 | 2.6 | 1.19 | 0.90 | 1.59 | 1.12 | 0.84 | 1.49 | 1.05 | 0.79 | 1.41 |
| Very high (Q5) | 89 | 2.2 | 1 |  |  | 1 |  |  | 1 |  |  |
|  |  |  |  |  |  |  |  |  |  |  |  |
| Unit decrease | 758 | 3.6 | **1.26** | **1.19** | **1.33** | **1.17** | **1.11** | **1.24** | **1.09** | **1.02** | **1.17** |
|  |  |  |  |  |  |  |  |  |  |  |  |
| **Average trust in public and political institutions** ^b^ | | | | | | |  |  |  |  |  |
| Very low (Q1) | 160 | 3.9 | 1.09 | 0.96 | 1.25 | 0.98 | 0.76 | 1.26 | 1.09 | 0.86 | 1.39 |
| Low | 161 | 3.9 | **1.16** | **1.01** | **1.33** | 1.07 | 0.83 | 1.38 | 1.06 | 0.84 | 1.34 |
| Medium | 162 | 3.9 | 1.08 | 0.94 | 1.24 | 1.09 | 0.85 | 1.40 | 1.12 | 0.89 | 1.42 |
| High | 134 | 3.2 | 0.98 | 0.85 | 1.13 | 0.91 | 0.70 | 1.19 | 0.9  7 | 0.76 | 1.24 |
| Very high | 141 | 3.5 | 1 |  |  | 1 |  |  | 1 |  |  |
|  |  |  |  |  |  |  |  |  |  |  |  |
| Unit decrease (Q5) | 758 | 3.6 | 1.05 | 0.99 | 1.11 | 1.01 | 0.95 | 1.07 | 1.03 | 0.97 | 1.08 |
|  |  |  |  |  |  |  |  |  |  |  |  |
| **OBJECTIVE** |  |  |  |  |  |  |  |  |  |  |  |
| **Fragmentation ^c^ index** ^c^ |  |  |  |  |  |  |  |  |  |  |  |
| Very low (Q1) | 57 | 2.5 | 1 |  |  | 1 |  |  | 1 |  |  |
| Low | 88 | 2.8 | 1.12 | 0.80 | 1.59 | 1.09 | 0.77 | 1.54 | 1.00 | 0.71 | 1.41 |
| Medium | 94 | 2.8 | 1.11 | 0.79 | 1.56 | 1.03 | 0.73 | 1.44 | 0.92 | 0.66 | 1.29 |
| High | 146  3.7 | 3.7 | **1.50** | **1.09** | **2.06** | 1.28 | 0.93 | 1.76 | 1.07 | 0.77 | 1.48 |
| Very high (Q5) | 373 | 4.6 | **1.89** | **1.41** | **2.53** | **1.49** | **1.11** | **2.01** | 1.29 | 0.92 | 1.80 |
|  |  |  |  |  |  |  |  |  |  |  |  |
| Unit increase | 758 | 3.6 | **1.19** | **1.12** | **1.26** | **1.12** | **1.05** | **1.19** | 1.07 | 0.99 | 1.16 |
|  |  |  |  |  |  |  |  |  |  |  |  |
| **Deprivation index** ^d^ |  |  |  |  |  |  |  |  |  |  |  |
| Very low (Q1) | 278 | 2.8 | 1 |  |  | 1 |  |  | 1 |  |  |
| Low | 161 | 3.4 | **1.24** | **1.02** | **1.51** | 1.13 | 0.93 | 1.38 | 1.09 | 0.89 | 1.34 |
| Medium | 112 | 4.7 | **1.71** | **1.37** | **2.14** | **1.41** | **1.12** | **1.77** | **1.36** | **1.07** | **1.72** |
| High | 64 | 5.1 | **1.89** | **1.43** | **2.49** | **1.55** | **1.17** | **2.05** | **1.43** | **1.07** | **1.91** |
| Very high (Q5) | 143 | 6.0 | **2.25** | **1.83** | **2.77** | **1.68** | **1.36** | **2.08** | **1.49** | **1.19** | **1.87** |
|  |  |  |  |  |  |  |  |  |  |  |  |
| Unit decrease | 758 | 3.6 | **1.23** | **1.17** | **1.29** | **1.14** | **1.09** | **1.20** | **1.11** | **1.06** | **1.17** |
|  |  |  |  |  |  |  |  |  |  |  |  |

^a^ Individually-adjusted: trust in residential area; Fully-adjusted: trust in residential area, sex, age, migrant status, relationship status, lone dwelling, residential moves, education, income, employment, population density

^b^ Individually-adjusted: average trust in public and political institutions; Fully-adjusted: average trust in public and political institutions, sex, age, migrant status, relationship status, lone dwelling, residential moves, education, income, employment, population density

^c^ Individually-adjusted: relationship status, lone dwelling, residential moves; Fully-adjusted: sex, age, migrant status, relationship status, lone dwelling, residential moves, education, income, employment, population density

^d^ Individually-adjusted: income, education; Fully-adjusted: sex, age, migrant status, relationship status, lone dwelling, residential moves, education, income, employment, population density

Q = quintile

## Sensitivity analysis using multiple imputation

***Methods:***

At baseline, covariates in our data were missing had up to 11.2% (Supplemental table 1). Missingness increased with each subsequent wave of follow-up, with 1.1% missing outcomes in 2007, 19.5% missing in 2010, and 39.5% missing in 2014. We used multiple imputation with chained equations (MICE) to replace missing values with imputed values to reduce potential bias. We assumed data were missing at random and imputed 50 datasets, which were combined using Rubin’s rules for analysis.

To impute missing data, we used variables included in the analytic models, including responses from earlier waves of SPHC to predict outcomes in the subsequent waves, as well as auxiliary variables from SPHC and the Swedish Registers. We conducted sensitivity analysis on several variables at the neighbourhood level to test if the assumption held that complete case analysis would generate similar results to those generated using multiple imputation (Supplemental table 6).

***Results:***

We found minor differences between the estimates that were generated using complete case analysis and imputed analysis. We assessed these differences to be small, particularly as the interpretation of the findings did not change between the two approaches. We chose to keep the complete case analysis as the primary analysis.

**Supplemental table 6 Neighbourhood-level factors and suicidal thoughts (unadjusted), complete case and imputed**

|  | **Complete case** | | | **Imputed** | | |  |
| --- | --- | --- | --- | --- | --- | --- | --- |
|  | OR | 95% | CI | OR | 95% | CI |  |
| **SUBJECTIVE** |  |  |  |  |  |  |  |
| **Trust in residential area** |  |  |  |  |  |  |  |
| Very low (Q1) | **1.57** | **1.38** | **1.78** | **1.56** | **1.37** | **1.76** |  |
| Low | **1.40** | **1.23** | **1.59** | **1.40** | **1.24** | **1.59** |  |
| Medium | **1.28** | **1.12** | **1.46** | **1.29** | **1.14** | **1.47** |  |
| High | 0.98 | 0.85 | 1.12 | 0.99 | 0.86 | 1.13 |  |
| Very high (Q5) | 1 |  |  | 1 |  |  |  |
|  |  |  |  |  |  |  |  |
| Unit increase | **1.13** | **1.10** | **1.17** | **1.13** | **1.10** | **1.16** |  |
|  |  |  |  |  |  |  |  |
| **Average trust in public and political institutions** | | | |  |  |  |  |
| Very low (Q1) | 1.09 | 0.96 | 1.25 | 1.09 | 0.95 | 1.24 |  |
| Low | **1.16** | **1.01** | **1.33** | **1.15** | **1.01** | **1.32** |  |
| Medium | 1.08 | 0.94 | 1.24 | 1.08 | 0.94 | 1.24 |  |
| High | 0.98 | 0.85 | 1.13 | 0.98 | 0.85 | 1.13 |  |
| Very high (Q5) | 1 |  |  | 1 |  |  |  |
|  |  |  |  |  |  |  |  |
| Unit increase | **1.03** | **1.00** | **1.06** | **1.03** | **1.00** | **1.06** |  |
| **OBJECTIVE** |  |  |  |  |  |  |  |
| **Fragmentation index** |  |  |  |  |  |  |  |
| Very low (Q1) | 1 |  |  | 1 |  |  |  |
| Low | 1.12 | 0.80 | 1.59 | 1.08  1.34  1.25  1.45 | 0.91 | 1.28 |  |
| Medium | 1.11 | 0.79 | 1.56 | 1.03 | 0.87 | 1.22 |  |
| High | **1.50** | **1.09** | **2.06** | **1.27** | **1.08** | **1.49** |  |
| Very high (Q5) | **1.89** | **1.41** | **2.53** | **1.46** | **1.26** | **1.69** |  |
|  |  |  |  |  |  |  |  |
| Unit increase | **1.19** | **1.12** | **1.26** | **1.11** | **1.08** | **1.14** |  |
| **Deprivation ^d^ index** ^d^ |  |  |  |  |  |  |  |
| Very low (Q1) | 1 |  |  | 1 |  |  |  |
| Low | 1.08 | 0.97 | 1.21 | 1.08  1.34  1.25  1.45 | 0.97 | 1.20 |  |
| Medium | **1.34** | **1.18** | **1.53** | **1.34** | **1.18** | **1.53** |  |
| High | **1.24** | **1.04** | **1.48** | **1.25** | **1.05** | **1.48** |  |
| Very high (Q5) | **1.47** | **1.29** | **1.67** | **1.45** | **1.28** | **1.65** |  |
|  |  |  |  |  |  |  |  |
| Unit increase | **1.10** | **1.07** | **1.14** | **1.10** | **1.07** | **1.13** |  |
